# Circulating endocannabinoids and genetic polymorphisms as predictors of posttraumatic stress disorder symptom severity: heterogeneity in a community-based cohort

**DOI:** 10.1038/s41398-022-01808-1

**Published:** 2022-02-01

**Authors:** Terri A. deRoon-Cassini, Carisa L. Bergner, Samantha A. Chesney, Nicholas R. Schumann, Tara Sander Lee, Karen J. Brasel, Cecilia J. Hillard

**Affiliations:** 1 Department of Surgery, Division of Trauma and Acute Care Surgery, Medical College of Wisconsin, Milwaukee, WI United States; 2 Comprehensive Injury Center, Medical College of Wisconsin, Milwaukee, WI United States; 3 Froedtert Hospital, Milwaukee, WI United States; 4 Queens Medical Center, Honolulu, HI United States; 5 Charlotte Lozier Institute, Arlington, VA United States; 6 Department of Surgery, Oregon Health and Science University, Milwaukee, WI United States; 7 Department of Pharmacology and Toxicology and Neuroscience Research Center, Medical College of Wisconsin, Milwaukee, WI United States

**Keywords:** Predictive markers, Human behaviour

## Abstract

The endocannabinoid signaling system (ECSS) regulates fear and anxiety. While ECSS hypoactivity can contribute to symptoms of established post-traumatic stress disorder (PTSD), the role of the ECSS in PTSD development following trauma is unknown. A prospective, longitudinal cohort study of 170 individuals (47% non-Hispanic Caucasian and 70% male) treated at a level 1 trauma center for traumatic injury was carried out. PTSD symptom assessments and blood were obtained during hospitalization and at follow-up (6–8 months post injury). Serum concentrations of the endocannabinoids *N*-arachidonoylethanolamine (AEA) and 2-arachidonoylglycerol (2-AG) were determined at both time points and selected genetic polymorphisms in endocannabinoid genes, including rs324420 in fatty acid amide hydrolase, were assessed. For the entire sample, serum concentrations of AEA at hospitalization were significantly higher in those diagnosed with PTSD at follow-up (*p* = 0.030). Serum concentrations of 2-AG were significantly, positively correlated with PTSD symptom severity at follow-up only in minorities (*p* = 0.014). Minority participants (mostly Black/African American) also demonstrated significant, negative correlations between serum AEA concentrations and PTSD symptom severity both measured at hospitalization (*p* = 0.015). The A/A genotype at rs324420 was associated with significantly higher PTSD symptom severity (*p* = 0.025) and occurred exclusively in the Black participants. Collectively, these results are contrary to our hypothesis and find positive associations between circulating endocannabinoids and risk for PTSD. Minority status is an important modulator of the association between endocannabinoids and risk for PTSD, suggesting that the ECSS contributes to risk most significantly in these individuals and the contextual factors related to these findings should be further explored.

## Introduction

Each year, approximately 2.5 million people in the United States are involved in a traumatic event that requires emergent care at a trauma center [1, 2]. Epidemiological studies indicate that over 10% of the general population has a diagnosis of post-traumatic stress disorder (PTSD) [3], and those who have experienced a traumatic injury are at greater risk for the disorder than the general population [4]. PTSD following injury is negatively related to the physical and mental quality of life after trauma and occurs in 25–40% of those traumatically injured [5]. Given these data, understanding factors that predict risk for the ultimate development of PTSD will inform better clinical approaches to the treatment of trauma that prevent PTSD.

Considerable evidence indicates that the endocannabinoid signaling system (ECSS) is stress-responsive and contributes to the regulation of anxiety and fear [6]. The ECSS includes the CB1 cannabinoid receptor (CB1R) and two endogenous ligands, *N*-arachidonoylethanolamine (AEA) and 2-arachidonoylglycerol (2-AG) [7]. CB1Rs are highly expressed throughout the brain, including regions involved in the processing of fear and formation of memories [8]. Brain ECSS subserves activity-dependent, retrograde synaptic signaling, thus plays a vital role in moment-to-moment synaptic activity [9]. Importantly, glucocorticoids [10, 11] and corticotropin-releasing factor [12] regulate brain concentrations of 2-AG and AEA, respectively; and many preclinical studies demonstrate that the ECSS is an important link between stress exposure and changes in synaptic activity, particularly in limbic brain regions [13].

Preclinical studies demonstrate that hypoactivity of the ECSS produces effects that are similar to symptoms of PTSD, including increased anxiety-related behaviors [14], deficient extinction of aversive memories [15], and sleep dysregulation [16]. On the other hand, the elevation of AEA-mediated CB1R activation through inhibition of fatty acid amide hydrolase (FAAH) is anxiolytic under aversive conditions [14]; promotes fear extinction, a component of coping and adaptation following traumatic stress [17]; and normalizes PTSD-like symptoms in a rat model [18]. In a clinical sample exposed to the 9/11 World Trade Center attacks 4–6 years prior, diagnosis of non-remitting PTSD was significantly related to reduced concentrations of circulating 2-AG compared to trauma-exposed non-PTSD individuals [19]. Taken together, these studies support the hypothesis that hypoactive ECSS could exacerbate while increased ECSS activity could alleviate PTSD symptoms.

Polymorphisms in human genes for proteins of the ECSS have been identified that support a role for the ECSS in emotional regulation [20]. In particular, polymorphisms in the CB1R gene (*CNR1*) are associated with anhedonia in individuals exposed to childhood abuse [21] and could interact with other genes to contribute to anxiety phenotypes [22]. A genetic polymorphism in *FAAH* (*rs324420*) influences amygdalar reactivity in response to threatening, fearful, and angry faces in healthy subjects [17, 23]. Carriers of the A allele at this locus exhibit quicker habituation of amygdala reactivity to threat and low scores on a personality trait of stress-reactivity [17] but, in individuals with anxiety disorders, A allele carriers are more prone to anxiety [24, 25].

Thus, there is emerging evidence that the state of activation of the ECSS could contribute to the symptoms of established PTSD. However, what is not known is the relationship of the ECSS to the risk for the development of PTSD. To address this gap, we assessed serum 2-AG and AEA concentrations soon after trauma in traumatically injured individuals and examined their correlations with PTSD diagnosis and symptom burden 6–8 months later, particularly for ethnic/racial minority patients and women, who have been shown to be at greatest risk for PTSD following trauma [26]. Our central hypothesis, based upon the preclinical literature, proposed that elevated serum concentrations of 2-AG and AEA at the time of trauma would be associated with reduced PTSD symptoms 6–8 months later. Similarly, we predicted that individuals with the minor allele at *rs324420* would exhibit less PTSD symptom severity. Given that several polymorphisms in the gene for the CB1R have been associated with altered risk for the development of psychopathology [13], we carried out exploratory studies of *CNR1* polymorphisms at *rs1049353*, *rs806371*, and *rs2180619* in association with risk for PTSD without a priori hypotheses.

## Methods and materials

### Participants and study design

This study employed a prospective, longitudinal design with two assessment points (hospitalization and six-eight months following injury “follow-up”). Two hundred seventy-eight adults hospitalized for traumatic injury were recruited for the study at one Midwestern Level 1 trauma center to provide adequate power to detect a moderate effect size; 170 participants completed the follow-up assessment (61.2% retention). Exclusion criteria included: (1) greater than mild traumatic brain injury, (2) non-English speaking, (3) unable to communicate, (4) intentional self-inflicted injury, and (5) detained by law enforcement while in the hospital. During hospitalization and after clinical stabilization (on average, 4 days post-trauma), participants completed questionnaires to self-report demographic information and PTSD symptom severity and completed a blood draw for endocannabinoid and genetic analyses. Information was gathered from the participant’s medical record, including injury severity score (ISS), mechanism of injury (MOI), time since last meal, time of day, height, weight, and contraceptive use. At the follow-up visit, participants completed symptom assessment measures, a diagnostic clinical interview for PTSD, and a blood draw for serum endocannabinoid analysis.

Consent was obtained for all subjects and the Institutional Review Board at the Medical College of Wisconsin approved all research procedures.

### PTSD severity and diagnostic assessment

PTSD symptom severity was assessed at hospitalization and at follow-up with the PTSD Checklist for *DSM-5* (PCL5), a 20-item self-report measure [27]. Items were summed to create a total severity score and a severity score for each of the four symptom clusters [avoidance, hyperarousal, reexperiencing, and negative alterations in mood and cognitions (NAMC)]. All PTSD symptom severity scores in the study are based on the PCL5.

The Clinician-Administered PTSD Scale (CAPS) was used to determine diagnosis at follow-up [28]. The CAPS, a semi-structured interview designed to assess the DSM-5 diagnostic features of PTSD, yields a categorical rating of PTSD diagnostic status and is the gold standard assessment for obtaining a diagnosis of PTSD, with excellent reliability and validity statistics [28]. The CAPS was administered and scored by trained mental health research personnel (inter-rater reliability kappa value of 1.00 for diagnosis in a random subsample of 10% of the interviews).

### Prior trauma exposure

Prior exposure to trauma has been shown to be a risk factor for PTSD [26]. Therefore, we included the life event checklist (LEC) to characterize exposure to trauma prior to the injury event (assessed at time 1) and between the injury event and 6 months (assessed at time 2). This is a 17-item questionnaire that assessed different types of trauma including exposure to a motor vehicle crash and a physical assault, with a greater score indicative of more trauma exposure [27].

### Biospecimen collection and analyses

Blood was collected into red top, serum collection tubes (BD Vacutainer tubes) and was allowed to clot at room temperature for about 30 min. Blood was centrifuged at 4400 rpm for 20 min at 4 °C; serum was removed and stored in 1.0 ml aliquots in plastic cryovials at −80 °C until analysis. 2-AG and AEA concentrations were determined in lipid extracts of serum using isotope dilution, liquid chromatography-mass spectrometry following previously published methods [29]. Plasma cortisol levels were determined using commercially available radioimmunoassay (RIA) kits. The sensitivity for cortisol assay was 57.5 pg/mL and no data lower than the minimum detection level were found. Based on manufacturer’s reporting, intra-assay precision varies from 7.3–10.5 and inter-assay precision varies from 8.6–13.4 for high-to-low cortisol levels.

### Genetic analysis

DNA was extracted from 5 ml whole blood using the Qiagen Gentra Puregene Blood Kit. Extracted DNA (10 ng) was genotyped using ThermoFisher Scientific Taqman SNP Genotyping Assays (FAAH*: rs324420* and CNR1: *rs806371*, *rs1049353*, and *rs2180619*) and Genotyping Master Mix according to the manufacturer’s instructions. Samples were analyzed on the QuantStudio 12 K Flex Real-Time PCR instrument and genotypes manually called using QuantStudio 12 K Flex Software v.1.2.2.

### Statistical analysis

Statistical analyses were conducted using IBM SPSS Statistics for Windows, Version 27 (IBM Corp., Armonk, N.Y., USA) and R (Version 1.2.5042). Demographic differences between the total number of enrolled participants and the sample of participants who completed the follow-up procedures were examined using appropriate statistical techniques. Pearson’s correlation coefficients and corresponding two-tailed significance *p* values are reported for bivariate correlational analyses. Multivariate correlation plots were created with the *corrplot* package in R for complete observations, using Spearman correlation coefficients. Correlation plot *p* values were adjusted for multiple comparisons with the “Holm” method. Regression analyses were utilized to model the interaction of gender and circulating AEA or 2-AG on PTSD symptom severity. One-way ANOVAs were employed to test for statistically significant differences in PTSD symptom severity between genetic variants and groups based on gender and minority status. Significance values of less than 0.05 were considered statistically significant and those less than 0.10 were considered trending significant. Code is available upon request.

## Results

### Demographics

Of the 278 enrolled participants, 170 completed study follow-up, and all analyses were conducted on this final sample. There were no statistically significant differences in gender, race, injury severity score, educational attainment, employment status, or relationship status between participants who completed the follow-up and those who did not (data not shown). However, age was significantly different between the groups, with participants who completed the study being older (µ = 42.8, *s* = 16.5) than participants who did not complete the study (µ = 35.2, *s* = 12.9). Demographics of the sample are included in Table 1.

**Table 1:** Descriptive statistics for sample and study variables.

Mechanism of injury	Percentage
MVC	32.4%
Fall	17.6%
GSW	16.5%
Stabbing	10.6%
MCC	8.2%
PSV	5.9%
Crush injury	4.7%
Recreational injury	1.8%
Other, assaultive	1.8%
Other, non-assaultive	0.6%
**Gender**	
Male	69.4%
**Race/Ethnicity**	
Caucasian	47.1%
Black American	44.1%
Hispanic/Latinx	7.6%
American Indian/Alaskan Native	1.2%
**Education**	
Completed at least some college	57.1%
**Employment status**	
Employed	65.9%
**Relationship status**	
In a committed relationship	61.8%
**Age**	
Mean (SD)	42.8 (16.5)
Range	18–89
**Study Variable**	Mean (SD)
PCL5 total severity score	
Hospitalization	18.86 (16.90)
6–8 month follow-up	20.55 (20.56)
Circulating 2-AG concentrations (nM)	
Hospitalization	668.37 (915.59)
6–8 month follow-up	91.26 (132.67)
Circulating AEA concentrations (nM)	
Hospitalization	2.53 (0.99)
6–8 month follow-up	2.37 (0.82)

*MVC* motor vehicle crash, *GSW* gunshot wound, *MCC* motor cycle crash, *PSV* pedestrian struck by vehicle, *PCL5* post-traumatic stress disorder checklist for DSM-5.

### Descriptive statistics

**Fig. 1:**
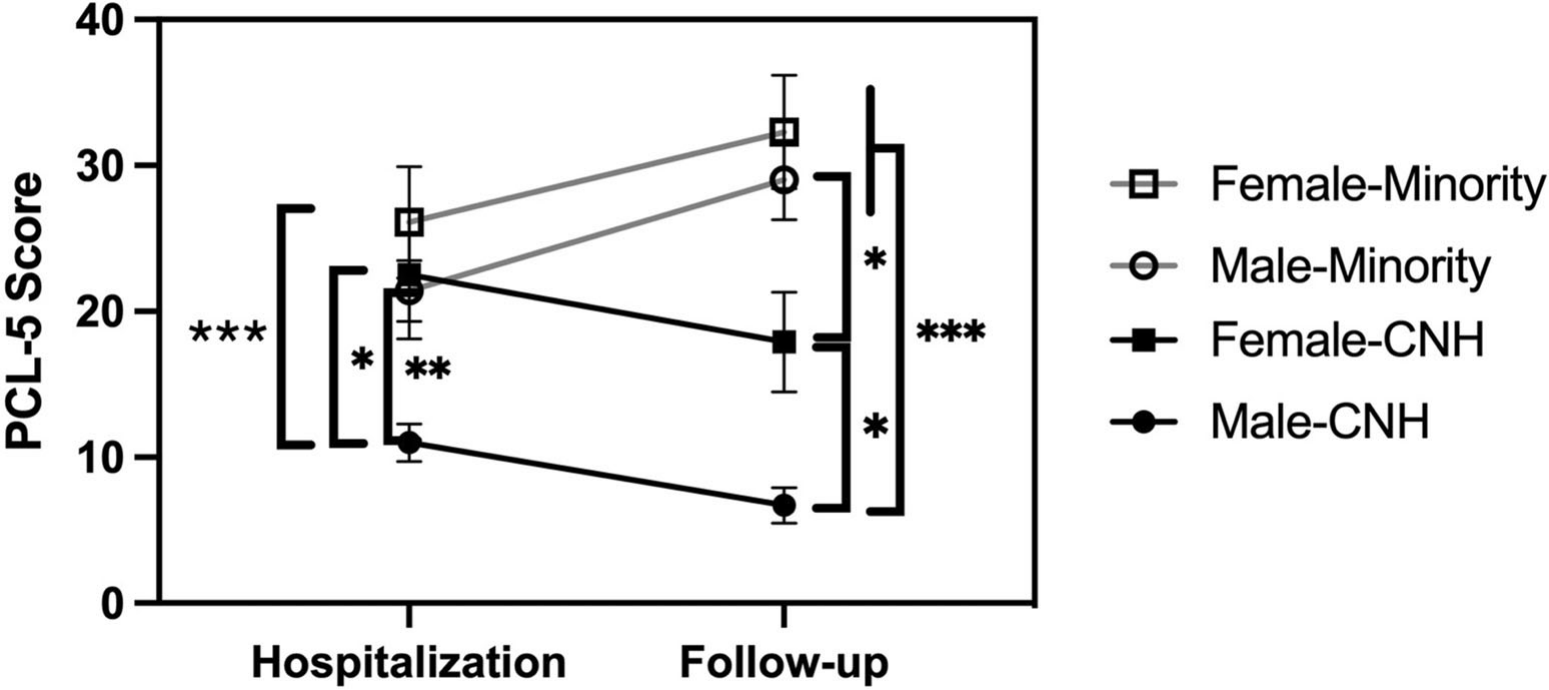
Total PCL5 scores were measured at hospitalization and follow-up, which occurred 6–8 months after discharge. The total cohort of 170 individuals was divided into subgroups based upon self-reported race, ethnicity, and sex. The minority subgroup included individuals endorsing African American, Black, Hispanic, and Native American. The number in each group is Caucasian, non-Hispanic (CNH) men (57); CNH women (23); minority men (61), and minority women (29). Symbols represent the mean; vertical lines represent the SEM. *p < 0.05; **p < 0.005, and ***p < 0.001 by Tukey’s HSD test.

At follow-up, 29.4% (*n* = 50/170) of participants met *DSM-5* criteria for PTSD, as assessed by the CAPS. Descriptive statistics of PTSD symptom severity (PCL5 scores) and circulating endocannabinoid concentrations at the time of hospitalization and follow-up are presented in Table 1. Compared to those who experienced a non-assaultive injury, those who experienced an assaultive injury reported significantly higher PTSD symptom severity both at the time of hospitalization for the injury, *t*(168) = 2.052; *p* < 0.05; mean difference = 5.82, and at follow-up, *t*(168) = 5.075; *p* < 0.001; mean difference = 16.26. While the PCL5 scores for the aggregate sample did not change significantly between hospitalization and follow-up, there were significant differences in the PCL5 scores among four demographic groups that could be established and maintain sufficient N: Caucasian race and non-Hispanic (CNH) men (*n* = 57), CNH women (*n* = 23), minority (those endorsing any of the following race or ethnicity categories: African American, Hispanic/Latino, and American Indian/Alaskan Native) men (*n* = 61) and minority women (*n* = 29). There were significant differences among the four groups in PTSD symptoms as measured by the PCL5 at both hospitalization (*F*(3,166) = 7.449, *p* = 0.000) and follow-up (*F*(3,166) = 21.562, *p* < 0.001) (Fig. 1). Tukey’s HSD post hoc tests revealed that the total PCL5 scores for CNH men were significantly lower at both time points than the scores for all other groups (at baseline: CNH men and CNH women *p* = 0.021; CNH men and minority men *p* = 0.003; CNH men and minority women *p* < 0.001; at follow-up: CNH men and CNH women *p* = 0.047; CNH men and minority men *p* < 0.001; CNH men and minority women *p* < 0.001). Additionally, the PCL5 scores for CNH and minority females were significantly different at follow-up (*p* = 0.018). It is important to note that CNH and minority participants had significantly different total scores on the LEC (*p* = 0.037) at baseline, with minority participants scoring higher on average (μ = 5.18) than CNH participants (μ = 4.43) for exposure to trauma, a finding that was replicated at the 6–8 month timeframe using the LEC (*p* = 0.034, μ = 1.46 vs μ = 0.54).

**Fig. 2:**
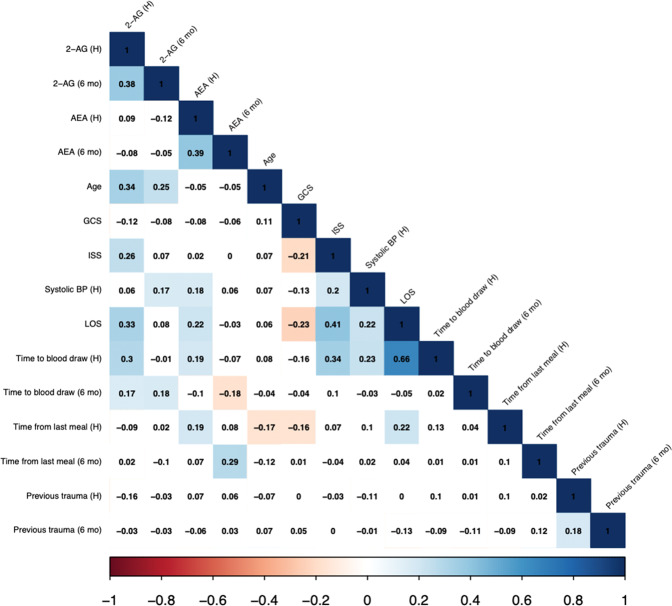
Correlation matrix of serum endocannabinoid concentrations at both time points; and selected demographic and clinical characteristics. 2-AG = 2-arachidonoylglycerol; AEA = *N*-arachidonoylethanolamine; H = hospitalization; 6 mo = 6–8 months follow-up visit; GCS = Glasgow Coma Scale; ISS = injury severity score; BP = blood pressure; LOS = length of stay in the hospital. Time to blood draw refers to the number of days from trauma to blood draw in hospital (H) and follow-up (6 mo); time from last meal is hours from last time eating to a blood draw in the hospital (H) and at follow-up (6 mo). Previous trauma (H) is trauma history prior to injury; and Previous trauma (6 mo) is trauma history between injury to follow-up assessment.

The mean concentration of 2-AG was very high in the samples taken during hospitalization (668 nM) compared to typical concentrations of 50–200 nM found in healthy volunteers [20]. Analysis of endocannabinoid values indicated a strong positive skew at hospitalization for 2-AG (2.39, SE = 0.19) and AEA (2.40, SE = 0.19), and for 2-AG at follow-up (4.06, SE = 0.20). Therefore, all four values were log-transformed prior to analysis. Log concentrations of both 2-AG and AEA were significantly, positively correlated between the two time points (Fig. 2; *p* < 0.001). There were no significant correlations between the two endocannabinoids at either time point.

Correlational analyses were used to test for associations between AEA and 2-AG concentrations and potentially confounding variables (Fig. 2). Age exhibited a significant, positive correlation with serum 2-AG at both time points (*p* < 0.001). At hospitalization, serum 2-AG was positively correlated with ISS (*p* < 0.001), LOS (*p* < 0.001), and time to blood draw (*p* < 0.001), all factors related to injury severity. AEA concentrations at hospitalization were significantly, positively associated with LOS (*p* = 0.007) and time to blood draw (*p* = 0.019) as well as systolic blood pressure (*p* = 0.031), but not with ISS (*p* = 0.776). AEA but not 2-AG concentrations at both time points were positively correlated with time since the last meal (baseline: *p* = 0.020; follow-up: *p* < 0.001), which is in accord with earlier findings that AEA concentrations rise in the fasted state [30]. Interestingly, AEA concentrations were positively correlated with body mass index (BMI) at follow-up (*p* < 0.005) but not at hospitalization (*p* = 0.62). 2-AG was not correlated with BMI at either time point.

### Circulating endocannabinoids and PTSD diagnosis

**Fig. 3:**
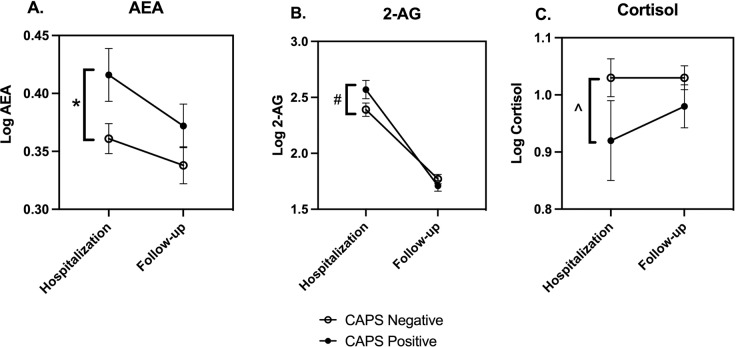
Endocannabinoid and cortisol concentrations at hospitalization and 6–8 months after injury in those with and without CAPS diagnosis of PTSD. PTSD was diagnosed at the follow-up visit using the CAPS; AEA (**A**), 2-AG (**B**), and cortisol (**C**) concentrations were determined in serum from blood samples collected at the time of hospitalization and at follow-up, which occurred 6–8 months following hospital discharge. Each data set was analyzed by two-way ANOVA and planned comparisons between PTSD positive and negative groups were made at each time point (i.e., hospitalization and follow-up). The ANOVA results are: AEA: Time *F*_(1,312)_ = 3.42, *p* = 0.066; PTSD *F*_(1,312)_ = 6.03, *p* = 0.015; Interaction *F*_(1,312)_ = 0.34, *p* = 0.56; 2-AG: Time *F*_(1,312)_ = 130, *p* < 0.0001; PTSD *F*_(1,312)_ = 0.86, *p* = 0.35; Interaction *F*_(1,312)_ = 3.43, *p* = 0.065; and cortisol: Time *F*_(1,315)_ = 0.60, *p* = 0.44; PTSD *F*_(1,315)_ = 4.03, *p* < 0.05; Interaction *F*_(1,315)_ = 0.60, *p* = 0.44. Symbols represent mean and vertical lines indicate SEM. *p < 0.05; #*p* = 0.08, and ^*p* = 0.09 by Sidak’s multiple comparison test.

When all study subjects are considered together, those diagnosed with PTSD at follow-up using CAPS had significantly higher concentrations of AEA (*p* = 0.030) and trending higher concentrations of 2-AG (*p* = 0.088) in their hospital blood sample than those negative for PTSD (Fig. 3A, B). Endocannabinoid concentrations at follow-up were not different between those with and without a PTSD diagnosis. As has been reported previously [31, 32], cortisol concentrations in the hospital blood sample trended significantly lower (*p* = 0.092) in those diagnosed with PTSD at follow-up (Fig. 3C). There were no differences in cortisol concentrations in the two diagnostic groups at follow-up.

### Circulating endocannabinoids and PTSD symptoms

The correlations among circulating concentrations of AEA and 2-AG and the PCL5 total and subscores at both time points for the entire sample were analyzed. In the combined sample, AEA concentrations at follow-up were positively correlated with total PCL5 (*p* = 0.046) and the arousal subscore (*p* = 0.017) both at hospitalization, as were AEA concentrations and PCL5 avoidance subscores (*p* = 0.032) both at follow-up. 2-AG concentrations at follow-up were significantly, negatively associated with avoidance subscores (*p* = 0.047) at hospitalization.

AEA concentrations at hospitalization were significantly related to change in PTSD symptom severity between hospitalization and follow-up (*p* = 0.032).

### Sex differences

Bivariate regression analyses were performed with an interaction term for sex and AEA or 2-AG concentrations at hospitalization or follow-up. The interaction term for sex and follow-up concentrations of AEA trended statistically significant (*p* < 0.10). The interactions for sex and 2-AG concentrations were not significant.

When the correlations between PCL5 scores and endocannabinoid concentrations were calculated in women alone (*n* = 52), AEA concentrations at follow-up were positively correlated with overall PTSD symptom severity (*p* = 0.002) at the same time, as well as with each symptom cluster (reexperiencing *p* = 0.024; avoidance *p* = 0.002; NACM *p* = 0.008; arousal *p* = 0.004). AEA concentrations at hospitalization were significantly correlated with follow-up avoidance symptoms (*p* = 0.038) and trended significantly correlated with follow-up NACM subscores (*p* = 0.081). There were no significant correlations between 2-AG concentrations and PCL5 scores when data from women alone were analyzed.

When the correlations between PCL5 scores and circulating concentrations of endocannabinoids were calculated in men alone (*n* = 118), the only significant relationships were a positive correlation between the arousal PCL5 subscore at hospitalization and AEA concentrations at follow-up (*p* = 0.030) and a negative correlation between the avoidance PCL5 subscore at hospitalization and 2-AG concentrations at follow-up (*p* = 0.030).

### Racial differences

For this analysis, the sample was divided into two groups: men and women endorsing Caucasian race without Hispanic ethnicity (*n* = 80; CNH) and those endorsing any of the following race or ethnicity categories: African American, Hispanic/Latino, and American Indian/Alaskan Native (*n* = 90; minority). In the analysis of the correlations in the minority group, significant, positive correlations were seen between 2-AG concentrations at hospitalization and all of the PCL5 measures at follow-up; the most significant correlation (*r* = 0.32) was with the reexperiencing subscore (*p* = 0.005). A different pattern was seen with AEA: concentrations were significantly negatively correlated with total PTSD symptoms (*p* = 0.15) and each of the subscores at hospitalization (reexperiencing *p* = 0.013; avoidance *p* = 0.006; NACM *p* = 0.048; arousal *p* = 0.015).

In men and women endorsing CNH race/ethnicity, significant negative correlations were found between 2-AG concentrations at follow-up and reexperiencing (*p* = 0.048) and avoidance PCL5 subscores (*p* = 0.015) at hospitalization. AEA concentrations at hospitalization were significantly, positively correlated with both the total PCL5 (*p* = 0.049) and arousal subscore (*p* = 0.018) at hospitalization.

### Genetic findings

Genotype at *rs324420* in FAAH and three SNPs in the gene for the CB1R (*rs806371*, *rs1049353*, and *rs2180619*) were analyzed in the context of PTSD symptom severity at hospitalization and follow-up in the total sample, men and women separately, and ethnic/racial minorities separately (Table 2).

**Table 2:** PCL5 symptom severity at hospitalization and follow-up based on genotype.

Gene SNP	Group	Allele (N)	Mean (SD) PTSD symptom severity hospitalization	Mean (SD) PTSD symptom severity follow-up
FAAH *rs324420*	All	A/A	21.85 (19.87)	34.47 (22.35)
		(20)		
		C/A	19.39 (15.31)	21.3 (19.67)
		(67)		
		C/C	18.12 (17.47)	16.83 (19.01)
		(81)		
PCL5 at Hospitalization: [*F*(2,165) = 0.407, *p* = 0.666]; PCL5 follow-up: [*F*(2,165) = 6.496, *p* = 0.002]				
	Men	A/A	16.63 (13.47)	33.00 (21.11)
		(16)		
		C/A	19.4 (15.71)	20.76 (19.68)
		(50)		
		C/C	13.88 (12.82)	11.34 (17.03)
		(50)		
PCL5 at hospitalization: [*F*(2,113) = 1.882, *p* = 0.157]; PCL5 follow-up: [*F*(2,113) = 8.760, *p* < 0.001]				
	Women	A/A	42.75 (29.41)	40.37 (29.65)
		(4)		
		C/A	19.35 (14.54)	22.88 (20.13)
		(17)		
		C/C	24.97 (21.62)	25.68 (18.94)
		(31)		
PCL5 at hospitalization: [*F*(2,49) = 2.192, *p* = 0.12]); PCL5 at follow-up: [*F*(2,49) = 1.224, *p* = 0.303]				
	Minorities	A/A	23.24 (20.83)	39.56 (20.17)
		(17)		
		C/A	23.34 (16.37)	29.24 (20.12)
		(38)		
		C/C	22.88 (17.94)	26.71 (21.7)
		(34)		
PCL5 at hospitalization: [*F*(2,86) = 0.006, *p* = 0.994]; PCL5 at follow-up: [*F*(2,86) = 2.251, *p* = 0.111]				
	African Americans	A/A	23.24 (20.83)	39.56 (20.17)
		17		
		C/A	24.53 (17.39)	28.47 (19.42)
		32		
		C/C	24.04 (19.29)	30.27 (21.86)
		26		
PCL5 at Hospitalization: [*F*(2,72) = 0.026, *p* = 0.974]; PCL5 follow-up: [*F*(2,72) = 1.715, *p* = 0.187				
CNR1 *rs806371*	All	G/G	20.13 (10.75)	31.00 (24.58)
		(8)		
		G/T	21.36 (17.39)	24.58 (21.45)
		(45)		
		T/T	18.1 (17.06)	18.48 (19.28)
		(115)		
PCL5 at hospitalization: [*F*(2,165) = 0.613, *p* = 0.543]; PCL5 at follow-up: [*F*(2,165) = 2.580*, p* = 0.079]				
	Men	G/G	21.17 (11.14)	24.17 (24.77)
		(6)		
		G/T	21.13 (16.68)	23.06 (22.28)
		(32)		
		T/T	14.45 (13.11)	16.03 (18.47)
		(78)		
PCL5 at hospitalization: [*F*(2,113) = 2.868, *p* = 0.061]; PCL5 at follow-up: [*F*(2,113) = 1.687, *p* = 0.190				
	Women	G/G	17.00 (12.73)	51.50 (6.36)
		(2)		
		G/T	21.92 (19.74)	28.31 (19.58)
		(13)		
		T/T	25.81 (21.53)	23.66 (20.18)
		(37)		
PCL5 at hospitalization: [*F*(2,49) = 0.299, *p* = 0.743]; PCL5 at follow-up: [*F*(2,49) = 1.994, *p* = 0.147]				
	Minorities	G/G	20.13 (10.75)	31.00 (24.58)
		(8)		
		G/T	25.38 (16.62)	32.55 (20.94)
		(29)		
		T/T	22.37 (19.14)	28.84 (20.87)
		(52)		
PCL5 at hospitalization: [*F*(2,86) = 0.394, *p* = 0.676]; PCL5 at follow-up: [*F*(2,86) = 0.291, *p* = 0.748]				
CNR1 *rs1049353*	All	A/A	16.67 (15.91)	16.83 (21.61)
		(6)		
		G/A	18.16 (17.89)	20.98 (20.54)
		(43)		
		G/G	19.52 (16.68)	20.81 (20.34)
		(199)		
PCL5 at hospitalization: [*F*(2,165) = 0.164, *p* = 0.849]; PCL5 at follow-up: [*F*(2,165) = 0.113 *p* = 0.893]				
	Men	A/A	12.75 (11.5)	12.00 (14.72)
		(4)		
		G/A	14.75 (16.11)	19.18 (21.47)
		(28)		
		G/G	17.45 (13.89)	18.43 (19.87)
		(84)		
PCL5 at hospitalization: [*F*(2,113) = 0.521, *p* = 0.595]; PCL5 at follow-up: [*F*(2,113) = 0.223, *p* = 0.801]				
	Women	A/A	24.50 (26.16)	26.50 (37.48)
		(2)		
		G/A	24.53 (19.83)	24.33 (18.92)
		(15)		
		G/G	24.49 (21.42)	26.53 (20.59)
		(35)		
PCL5 at hospitalization: [*F*(2,49) = 0.000, *p* = 1.000]; PCL5 at follow-up: [*F*(2,49) = 0.060, *p* = 0.941]				
	Minorities	A/A	43.00 ()	53.00 ()
		(1)		
		G/A	26.64 (21.63)	41.91 (19.58)
		(11)		
		G/G	22.39 (17.1)	28.28 (20.78)
		(77)		
PCL5 at hospitalization: [*F*(2,86) = 0.915, *p* = 0.405]; PCL5 at follow-up: [*F*(2,86) = 2.711, *p* = 0.072]				
CNR1 *rs2180619*	All	A/A	15.22 (14.7)	13.73 (15.64)
		(37)		
		G/A	19.28 (17.55)	22.3 (21.17)
		(86)		
		G/G	21.39 (17.13)	23.15 (21.02)
		(46)		
PCL5 at hospitalization: [*F*(2,166) = 1.407, *p* = 0.248]; PCL5 at follow-up: [*F*(2,166) = 2.851, *p* = 0.061]				
	Men	A/A	14.35 (13.99)	12.35 (15.49)
		(26)		
		G/A	17.53 (14.76)	21.27 (21.51)
		(60)		
		G/G	16.32 (14.07)	17.65 (19.45)
		(31)		
PCL5 at hospitalization: (*F*(2,114) = 0.447, *p* = 0.641); PCL5 at follow-up: [*F*(2,114) = 1.868*, p* = 0.159]				
	Women	A/A	17.27 (16.79)	17.00 (16.23)
		(11)		
		G/A	23.31 (22.56)	24.67 (20.57)
		(26)		
		G/G	31.87 (18.55)	34.53 (20.08)
		(15)		
PCL5 at hospitalization: [*F*(2,49) = 1.715*, p* = 0.191]; PCL5 at follow-up: [*F*(2,49) = 2.636, *p* = 0.082]				
	Minorities	A/A	21.00 (24.99)	34.60 (16.41)
		(5)		
		G/A	21.66 (17.79)	29.35 (21.9)
		(53)		
		G/G	25.25 (16.79)	30.44 (20.62)
		(32)		
PCL5 at hospitalization: [*F*(2,87) = 0.434, *p* = 0.649]; PCL5 at folow-up: [*F*(2,87) = 0.149*, p* = 0.862]				

PCL5 is the total symptom severity score for the PTSD checklist for the DSM-5.

For FAAH (*rs324420*), individuals homozygous for the minor allele (A/A) had significantly higher PTSD symptom severity at follow-up than those heterozygous (*p* = 0.025) or with the C/C genotype (*p* = 0.001). This result appeared to be driven by results from men in the sample, as A/A women had a very high PTSD symptom burden at hospitalization which did not increase at follow-up. Concentrations of circulating AEA at hospitalization were significantly higher for those with the A/A genotype compared to those with either the C/C (*p* = 0.013) or C/A (*p* = 0.039) genotype and remained higher at follow-up for those with A/A compared to C/C genotype (*p* = 0.001). These findings were found at both time points in men (hospitalization, *p* = 0.013; follow-up, *p* = 0.018) and racial minorities (hospitalization, *p* = 0.010; follow-up, *p* = 0.021). There was a trend for serum AEA concentrations to be higher in the A/A variant group compared to C/C in women at follow-up only (*p* = 0.089). To determine if the development of PTSD and its relationship to FAAH was mediated by AEA, we conducted a bootstrapping method using the SPSS Process Macro and found that the indirect effect of AEA at baseline was not statistically significant (B = −0.24 SE = 0.56, 95% CI [−1.45, 0.80]).

Genotype at *rs806371* in *CNR1* exhibited a trending association with PTSD symptom severity at follow-up, with the rare allele (G) being associated with increased symptoms. In men, PTSD symptom severity at hospitalization but not follow-up trended significantly different with genotype at *rs806371*. Interestingly, individuals with at least one G at *rs806371* and at least one A at *rs323320* in *FAAH* had a significantly higher mean PTSD total score at follow-up (28.6 ± 22.4; *n* = 28) compared to those homozygous for the common alleles at both loci (14.2 ± 17.7; *n* = 57) (*p* = 0.002).

Individuals with the G/G allele at CNR1 *rs2180619* trended to exhibit higher PTSD symptom severity at follow-up compared to those with at least one copy of A (*p* = 0.061), with women, in particular, demonstrating a trending significant difference in G/G compared to A/A (*p* = 0.073).

When considering the CNR1 SNP at *rs1049353*, no significant differences in PTSD symptom severity were found at either time point for the total sample, or men and women separately. Minorities with the A/A allele, however, trended greater PTSD symptom severity at follow-up (*p* = 0.072), although post hoc analyses were not able to be run due to the small group sample size in A/A.

## Discussion

The overall purpose of this study was to explore and describe the associations between measures of endocannabinoid system function and post-traumatic stress disorder symptoms following injury in a community population. Our community study sample was relatively large (170 subjects) and diverse, as just over 50% were a racial or ethnic minority and 70% of the sample were male. The types of injury sustained were also diverse, just over 25% experienced an assaultive type injury and about 40% were injured in a motor vehicle accident. There was a striking difference in susceptibility for an increase in PTSD symptoms in the 6–8 months following the injury between the CNH and minority groups, with the former showing no increase in symptoms and the latter a significant increase. Thirty percent of the sample met the criteria for PTSD at 6–8 months, with minority women having the greatest PTSD symptom severity at this time point.

Our first objective was to explore the relationships between endocannabinoid concentrations at the time of hospitalization and PTSD symptoms 6–8 months later. Individuals with PTSD symptoms that persist beyond 6 months after injury are considered to have non-remitting PTSD, a condition which significantly reduces the quality of life [33]. When the data from all participants are considered together, individuals diagnosed with PTSD six-eight months after traumatic injury had significantly higher circulating AEA and a trend toward an increase in circulating 2-AG concentrations at hospitalization than those without a PTSD diagnosis. Importantly, circulating AEA concentrations at hospitalization were significantly, positively correlated with an increase in PTSD symptoms between hospitalization and 6–8 months follow-up, suggesting that elevated AEA at the time of trauma is associated with increased risk for the development of PTSD over time. When the data from men and women were considered separately, the associations between AEA concentrations at hospitalization and PCL5 scores at follow-up were more robust in women. Cortisol concentrations measured in the same blood samples trended to be lower in those who were diagnosed with PTSD at 6–8 months, which is consistent with some earlier studies particularly when the individuals had been subjected to early-life adversity [34]. Importantly, these findings suggest that the positive correlations between peritraumatic endocannabinoids and PTSD diagnosis at follow-up are not explained by higher cortisol concentrations evoked by the trauma.

The relationship between PTSD severity at follow-up and 2-AG concentrations at hospitalization was stronger and reached statistical significance when the data from ethnic and racial minorities were examined separately. In racial minority subjects only, greater 2-AG at hospitalization was significantly related to PTSD symptom severity at follow-up, with these findings particularly driven by the reexpriencing and avoidance subgroups of PTSD symptoms.

While the clear rationale for these differences based on sex and race is beyond the scope of this study, it is clear that the endocannabinoid stress response is not homogenous in humans, particularly in the acute aftermath of trauma. Further work will need to include an investigation of biologically based sex and racial differences and contextual factors that affect stress systems in the acute aftermath of a trauma. For example, recent work by our group has found that experiences of discrimination predict PTSD after trauma beyond acute stress [35]. Thus, it is possible that chronic stress prior to trauma, from such experiences as discrimination, exposure to community violence, and resource deprivation may alter the endocannabinoid system such that the endocannabinoid response to trauma is different compared to someone living in less chronically stressful conditions. Other findings suggest that prior trauma exposure impacts biologically based stress responding in the acute aftermath of a subsequent trauma, for instance, related to HPA axis functioning and the release of cortisol [34]. Importantly, manipulation of endocannabinoid signaling has been proposed as a potential target for intervention in PTSD [6] but further research on sex and race differences is warranted to inform such interventions.

Circulating AEA concentrations at hospitalization were associated most strongly with follow-up symptoms of avoidance and also trended to be associated with arousal. On the other hand, circulating 2-AG at hospitalization was most strongly associated with the reexperiencing symptom cluster at follow-up. The nonoverlapping associations of each endocannabinoid with PTSD symptoms suggest that they contribute differently to the plasticity that occurs between the time of injury and the development of non-remitting PTSD. This observation is in accord with the preclinical literature demonstrating that AEA and 2-AG have both overlapping and different roles in the regulation of amygdalar signaling [36]. We postulate that the stress of the injury mobilizes both endocannabinoids and that they contribute differently to changes in emotional circuitry in a manner that is modulated by sex and other factors.

Importantly, these findings indicate that high endocannabinoid tone in the peritraumatic period is associated with increased risk for the development of non-remitting PTSD, rather than reduced risk as originally hypothesized based upon studies showing that circulating endocannabinoids are lower in individuals with chronic PTSD [19, 37] and that an increase is associated with improved symptoms [38]. Additionally, a wealth of preclinical literature supports the notion that reduced endocannabinoid tone mirrors the symptoms of chronic PTSD [14–16]. A recent study examining proinflammatory cytokines in blood harvested within four hours of a traumatic injury found that low concentrations of interferon-gamma (IFNγ) and tissue necrosis factor-alpha (TNFα) were associated with increased chronic PTSD [39]. Like the present results, these cytokine findings are seemingly incongruent with data that proinflammatory cytokines are consistently found to be positively associated with chronic PTSD [40]. Thus, responses to trauma are dynamic and protective factors in the peritraumatic period could be associated with worsened symptoms in chronic PTSD.

While a recent study in rats found inhibition of FAAH after a trauma inhibited the development of long-term symptoms of anxiety and fear [41], other studies have found high concentrations of AEA facilitate affective, negative memories of the trauma [42]. Possibly, high concentrations of AEA contribute to PTSD through the development of negative emotional memory formation which in turn may lead to difficulty with fear extinction [43], a core component of PTSD [44]. In support of this hypothesis, Morena, Hill, and colleagues have provided evidence in rats that high FAAH activity in the amygdala is associated with low AEA concentrations and reduced HPA axis activation, anxiety, and expression of conditioned fear [45]. They suggest that, in the absence of stress, AEA tonically activates CB1 receptors on GABAergic neurons, resulting in suppression of this inhibitory input. Stress, which has been shown to increase FAAH activity and reduce AEA, would disinhibit the GABAergic neurons, resulting in suppression of amygdala activity.

Importantly, the current and previous findings suggest that role of AEA may change over time following trauma. Given that clinical studies also find that individual trajectories of PTSD symptoms emerge after trauma [5, 34], further work should elucidate the associations of trajectories in AEA concentrations with symptom change across time.

Our second objective was to examine the associations between circulating endocannabinoid concentrations and PTSD symptoms determined at the same time. Earlier studies addressing this question have provided conflicting results. In a study of individuals with long-standing PTSD following the World Trade Center attack, circulating 2-AG concentrations were lower in those with PTSD than trauma-exposed controls and AEA concentrations were negatively correlated with intrusive thoughts of the trauma [19]. On the other hand, a study of individuals with severe PTSD (mean CAPS scores of 95), found that both AEA and 2-AG were significantly elevated in individuals with PTSD compared to trauma-exposed individuals without PTSD [46]. In the current study, the strongest associations between contemporaneous measures of circulating endocannabinoids and PTSD severity were seen when the sample was stratified by sex or minority status. In women, AEA concentrations were strongly, positively correlated with overall PTSD symptom severity and with each symptom cluster individually at follow-up. These findings are contrary to some preclinical work suggesting that higher CNS AEA is related to reduced anxiety-like behavior [14], startle response, and extinction retention central to recovery following trauma [18]. However, inhibition of FAAH in female but not male rats was associated with impaired extinction of fear memory through a non-CB1 receptor mechanism [47], suggesting, as the current study does, that the relationship of FAAH and AEA to the risk for development of PTSD is sex-dependent.

Conversely, AEA concentrations and overall PTSD symptom severity were negatively correlated at hospitalization in individuals endorsing Black and American Native race and Latinx ethnicity. Among the individual symptom clusters, avoidance was most highly negatively correlated with AEA and significant, negative correlations were seen between AEA and reexperiencing and arousal, suggesting lower AEA concentrations are related to higher PTSD symptom severity in this subgroup at the time of injury. It is interesting that these associations did not emerge in the data obtained at follow-up. Our findings in ethnic and racial minorities may be an indication of ongoing sociocultural stress which has shown to increase the risk for PTSD [48], particularly in those living in urban environments. Our sample in this study was predominantly urban and further research is warranted to understand how ongoing cultural stress compounds outcomes following trauma, potentially through weathering effects as has been seen with other biomarkers such as allostatic load [49].

The lack of associations in the current study between PTSD symptoms and circulating 2-AG concentrations measured concurrently is contrary to findings that those with chronic PTSD have lower [19] or higher [46] circulating 2-AG concentrations as well as less robust recruitment of 2-AG in response to psychological or physical stress [38, 50]. It is possible that 2-AG has a more significant role in chronic stress management such that depletion of 2-AG after sustained periods of stress, such as having chronic PTSD, creates a vulnerability to ongoing stress.

Our third objective was to explore the contribution of polymorphisms in the genes for FAAH and CNR1 to the risk of developing PTSD. Genotype at a well-studied SNP in FAAH (*rs324420*) was significantly related to PTSD severity at follow-up but not hospitalization when the entire cohort was compared. Specifically, individuals homozygous for the minor allele A/A at this location experienced greater PTSD symptom severity 6–8 months following injury compared to individuals with at least one copy of the major allele (C). This relationship was most pronounced in men. Interestingly, women with the A/A genotype had high PCL5 scores at hospitalization (42 compared to 22 in the entire sample) although this enrichment did not reach statistical significance. There was no significant relationship between FAAH genotype and PTSD symptoms at either time point in minorities. As has been shown previously [29], individuals with A/A also had significantly greater circulating AEA concentrations. Thus, these results are consistent with our finding that circulating AEA concentrations are positively associated with PTSD severity. This finding is in contrast to some studies that have found individuals homozygous for the A allele exhibit a reduction in reactivity to stress and ultimate protection from negative emotional responses to stress exposure [29, 51]. However, other studies have found that the interaction of the minor allele and early-life adversity increases the risk for anxiety and depression later in life. Prior life trauma is a risk factor for developing subsequent PTSD [26] and the effect of prior trauma [52] coupled with the minor allele at this locus could create further vulnerability to PTSD. Despite previous work suggesting that SNPs in CB1R genes contribute to risk for pathology in adulthood following childhood trauma exposure, significant findings did not emerge related to the *CNR1* polymorphisms at *rs1049353* and *rs2180619* and PTSD diagnosis. There was a trending association of *rs806371* genotype with PTSD symptom severity at follow-up in the entire sample, an association that did not persist in the smaller sex and minority groups. The *rs806371* minor variant (G) is associated with reduced expression of the CB1R [53]. Individuals with an A at *rs324420* in FAAH and G at *rs806371* exhibited a significantly higher PTSD severity at follow-up than those homozygous for the more common allele at each locus. This suggests the hypothesis that high AEA (resulting from low FAAH) and reduced CB1R expression can synergize to enhance the risk for PTSD following traumatic injury.

An important note related to our study findings is the validity of the utilization of circulating endocannabinoid levels to characterize a system that is largely CNS activated. The circulating concentrations of 2-AG in the samples obtained at hospitalization were very high (mean of nearly 700 nM) compared to concentrations from healthy controls (typically 60–200 nM) [20, 54] and in the samples obtained at follow-up (90 nM). Several other studies have shown that acute trauma and injury are associated with high 2-AG concentrations [55, 56]. While AEA concentrations were not abnormally high, they trended to be correlated with the 2-AG concentrations at hospitalization even though the biochemical mechanisms for their synthesis differ [7]. These data are consistent with the notion that exposure to a very strong stressor mobilizes both 2-AG and AEA in concert as the body moves further away from homeostasis. Endocannabinoids in circulation can arise from various sources, including the CNS but also peripheral tissues and blood cells, and therefore can be considered integration of endocannabinoid tone throughout the body [30]. It is difficult to assess the relationship between CNS endocannabinoid signaling and the concentrations of endocannabinoids in circulation in humans. Studies in mice treated with an inhibitor of monoacylglycerol lipase found that circulating concentrations of 2-AG were very significantly associated with brain concentrations of 2-AG [36]. Recent data in humans using PET imaging to assess the amount of FAAH enzyme while simultaneously measuring circulating AEA concentrations found a negative correlation between CNS FAAH levels and AEA concentrations in the circulation [57]. Both of these support the hypothesis that CNS endocannabinoid tone can influence circulating concentrations of 2-AG and AEA. Finally, there is a wealth of data showing significant associations and correlations between circulating endocannabinoid concentrations and measures of CNS function [13, 30], further supporting the contention that circulating endocannabinoid concentrations are a reflection of the tone of CNS endocannabinoid signaling. These findings support the hypothesized role of the endocannabinoid system as a component of the stress response system [13].

Our findings need to be considered within the context of study limitations, particularly as our results are novel and generally contradictory to preclinical work. The blood draws completed with participants were not standardized with respect to time of day or food intake, for example, which have been shown to influence circulating endocannabinoid concentrations in other studies [30]. This suggests that circadian changes, time since the last meal, and time since trauma could influence the endocannabinoid measurements. Second, while this study enrolled 170 subjects, sample size differences for allelic expression in the genetic studies limits findings, and future larger studies are needed to better understand the roles of the SNPs studied in risk for PTSD.

Our data support hypotheses that the endocannabinoid system is stress-responsive and contributes to the effects of stress on emotional processing. Our study findings suggest that endocannabinoid signaling at the time of severe stress sets in motion changes in the brain that potentiate the likelihood of non-remitting PTSD many months later. These data also indicate that the specifics of the relationship between the endocannabinoids and PTSD risk are modulated by sex, genotype, and possibly prior trauma exposure. The clinical course of PTSD is diverse [58] and these results suggest that differences in the endocannabinoid response are a contributor to this diversity. Further work is needed to continue to discover how the endocannabinoid system is related temporally to pathology risk following trauma to refine our understanding of when and how the endocannabinoid system can be targeted for intervention to prevent PTSD development.
